# Monogenic Retinal Diseases Associated With Genes Encoding Phototransduction Proteins: A Review

**DOI:** 10.1111/ceo.14511

**Published:** 2025-02-27

**Authors:** Wendy M. Wong, Omar A. Mahroo

**Affiliations:** ^1^ Institute of Ophthalmology, University College London London UK; ^2^ NIHR Biomedical Research Centre at Moorfields Eye Hospital and the UCL Institute of Ophthalmology London UK; ^3^ Centre for Innovation & Precision Eye Health, Yong Loo Lin School of Medicine National University of Singapore Singapore Singapore; ^4^ Department of Ophthalmology, National University Hospital National University Health System Singapore Singapore; ^5^ Section of Ophthalmology, King's College London St Thomas' Hospital Campus London UK; ^6^ Physiology, Development and Neuroscience University of Cambridge Cambridge UK; ^7^ Department of Translational Ophthalmology Wills Eye Hospital Philadelphia Pennsylvania USA

**Keywords:** cone photoreceptors, inherited retinal disease, phototransduction, retina, rod photoreceptors

## Abstract

Phototransduction, the process by which captured photons elicit electrical changes in retinal rod and cone cells, represents the first neuronal step in vision and involves interactions between several highly specialised proteins. Pathogenic variants in genes encoding many of these proteins can give rise to significant vision impairment, accounting for a substantial portion of inherited retinal disease. Such genes include *RHO*, *OPN1LW*, *OPN1MW*, *GNAT1*, *GNAT2*, *GNB3*, *PDE6A*, *PDE6B*, *PDE6G*, *PDE6C*, *PDE6H*, *CNGA1*, *CNGB1*, *CNGA3*, *CNGB3*, *GRK1*, *SAG*, *ARR3*, *RGS9*, *RGS9BP*, *GUCY2D*, *GUCA1A* and *SLC24A1*. Many of these conditions have distinct mechanisms and clinical features. They follow several modes of inheritance (including in one case digenic, or tri‐allelic, inheritance). Some conditions also entail myopia. Rod and cone phototransduction will be outlined, followed by the discussion of diseases associated with these genes. Some phenotypic features will be highlighted as well as their prevalence in a large genotyped inherited retinal disease cohort.

## 1 Introduction

A feature of interest in monogenic retinal diseases is that the majority appear to primarily affect photoreceptors. Photoreceptors represent the first neurons in the visual pathway and are highly specialised, expressing a subset of genes distinct from most other cell types in the body. Thus, pathogenic variants in any one of a large number of genes will bring about diseases selectively affecting photoreceptors, with minimal effects on other cell types. Post‐receptoral retinal neurons share greater similarity with other neurons in the central nervous system, and so it might be expected that several genetic diseases that might affect these neurons could have a primary neurological phenotype in which any visual impairment might be less prominent.

**FIGURE 1:**
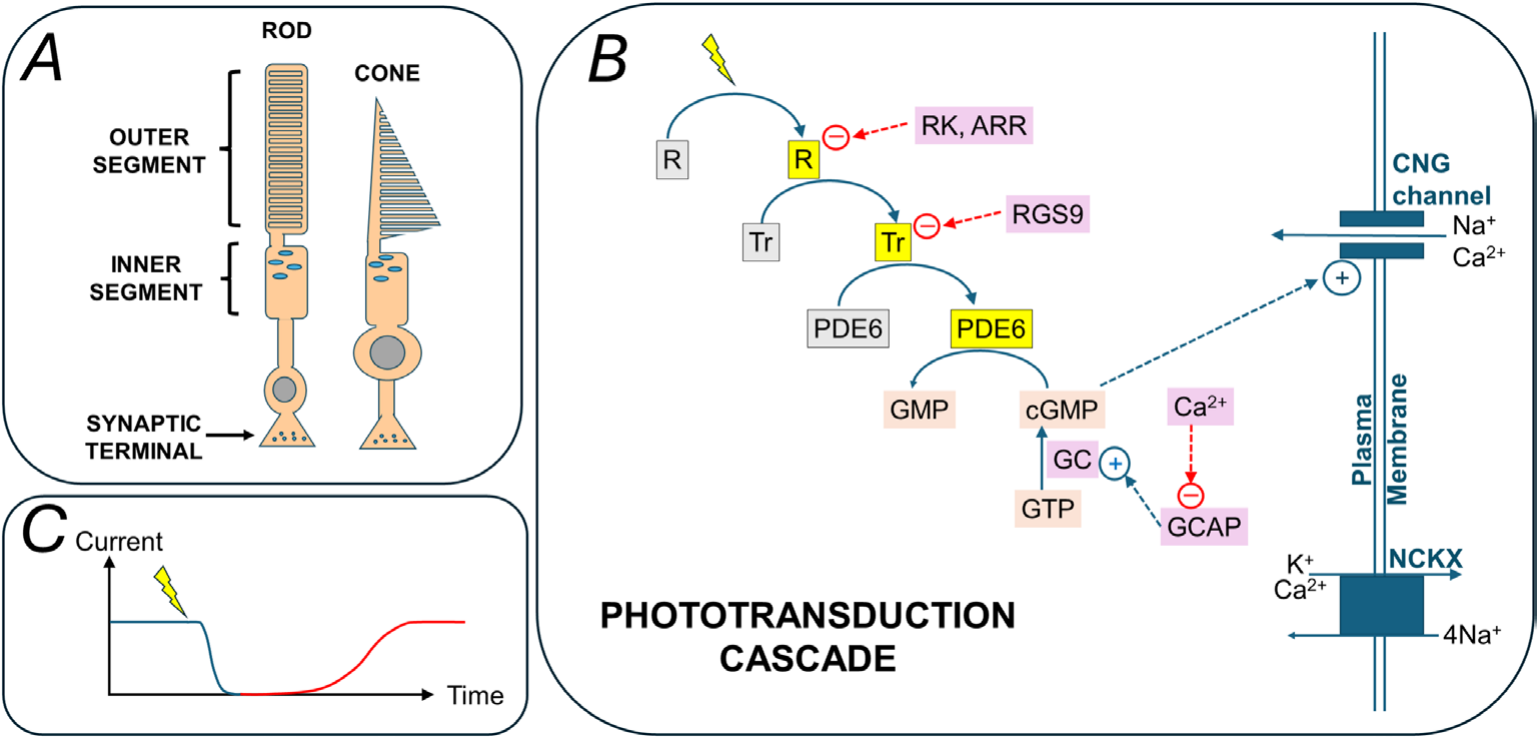
Phototransduction in rods and cones. (A) Schematic of a rod and a cone photoreceptor. Photoreceptors are highly specialised cells containing an outer segment composed of many discs (in rods) or sacs (in cones) which contain proteins of the phototransduction cascade. (B) Schematic of the phototransduction cascade. Rhodopsin (R in the diagram; in cones, this would be L‐, M‐ or S‐opsin) is activated by light, and in turn activates several transducin molecules (Tr). Activated transducin activates phosphodiesterase‐6 (PDE6), which hydrolyses cyclic guanosine monophosphate (cGMP) to guanosine monophosphate (GMP). cGMP maintains the open state of cation channels in the outer segment plasma membrane (hence these are termed cyclic nucleotide‐gated [CNG] channels). Closure of the CNG channels, due to the reduction in outer segment cGMP concentration, reduces entry of sodium and potassium ions, shutting off the inward current (depicted in C), causing the cell to hyperpolarise and reduce glutamate release at the synapse with bipolar cells. Each of these activation stages in the light response has shut‐off mechanisms, some of which are also depicted. Rhodopsin's activity is quenched through phosphorylation by rhodopsin kinase (RK) and capping by arrestin (ARR). Transducin's activity is regulated by Regulator of G‐protein signalling 9 (RGS9). cGMP levels are replenished by conversion from guanosine triphosphate (GTP) by guanylyl cyclase (GC), which itself is activated by GC activating protein (GCAP). GCAP is inhibited by calcium ions; the reduction in intracellular calcium secondary to cGMP channel closure relieves inhibition of GCAP, hence hastening the generation of cGMP and re‐opening of the channels. Channel reopening restores the inward current (as shown in C) allowing the cell to recover from hyperpolarisation. Also in the plasma membrane are sodium/calcium potassium exchangers (NCKX), enabling calcium to continually exit the cell. Relevant genes discussed in this review encode many of the proteins shown. R, rhodopsin, is encoded by *RHO*, and the cone opsins by *OPN1LW*, *OPN1MW* and *OPN1SW*. The alpha subunit of transducin, Tr, is encoded by *GNAT1* in rods and *GNAT2* in cones. *GNB3* encodes the beta subunit. *PDE6A*, *PDE6B* and *PDE6G* encode subunits of PDE6 in rods, whilst *PDE6C* and *PDE6H* encode subunits of cone PDE6. CNG channel subunits are encoded by *CNGA1* and *CNGB1* in rods and by *CNGA3* and *CNGB3* in cones. In rods, rhodopsin kinase and arrestin are encoded by *GRK1* and *SAG*, respectively; in cones the relevant genes are *GRK7* and *ARR3*. The RGS9 protein is encoded by the *RGS9* gene and its binding protein by *RGS9BP*. *GUCY2D* encodes guanylyl cyclase (GC) and its activating protein (GCAP) is encoded by *GUCA1A*. NCKX is encoded by *SLC24A1* in rods and *SLC24A2* in cones. (C) Schematic illustrating the reduction (and subsequent recovery) in current flowing into the photoreceptor outer segment after a light flash. Dimmer flashes cause less reduction; brighter flashes cause greater reduction and recovery takes longer. With light adaptation, recovery occurs more quickly.

One highly specialised process occurring within photoreceptors, which defines their function, is phototransduction, the process by which light photons are captured and bring about changes in the cell's membrane potential. The phototransduction cascade involves key steps, whose kinetics are highly regulated and determine the overall sensitivity of human vision in different light environments. Figure 1 summarises the cascade within rod and cone photoreceptors, highlighting the key proteins involved. Some of these are specific to rods or cones, whilst others are shared. The cascade in rods and cones will be described in Section 2. Sequence variants in the genes encoding these proteins can give rise to retinal disease, sometimes with distinct clinical features. Relevant disease‐associated genes to be discussed are listed in the legend to Figure 1 and are summarised in Table 1. These conditions will be described in Sections 3, 5. In Section 6, some phenotypic features will be highlighted (including aspects of imaging and electrophysiology). Section 7 will quantify numbers of affected individuals within a large UK‐based genotyped inherited retinal disease cohort who have disease associated with phototransduction genes and also give the most prevalent pathogenic variant for the five most frequently encountered genes in that cohort. Section 8 will briefly list some gene‐specific therapies on the horizon. The concluding section of this review will touch on the potential for insights to be afforded into more common, non‐monogenic eye diseases.

**TABLE 1:** Genes involved in phototransduction and associated inherited retinal diseases.

Process	Gene	Location	Encoded protein	Mode of inheritance	Range of phenotypes (MIM number)
Activation	*RHO*	3q22.1	Rhodopsin	Autosomal dominant	RP; sector RP (613731); stationary night blindness (610445)^a^
				Autosomal recessive	RP (613731)^a^
	*OPN1LW/OPN1MW*	Xq28	Long‐wave/medium‐wave sensitive cone opsin	X‐linked	Protanomaly/deuteranomaly Protanopia (303900)/deuteranopia (303800) Blue cone monochromacy (303700)^b^ Bornholm eye disease (300843)^b^
	*OPN1SW*	7q32.1	Short‐wave sensitive cone opsin	Autosomal dominant	Tritanopia (190900)
	*GNAT1*	3p21.31	Alpha subunit of rod transducin	Autosomal dominant	Stationary night blindness (610444)^a^
				Autosomal recessive	Stationary night blindness (616389)^a^
	*GNAT2*	1p13.3	Alpha subunit of cone transducin	Autosomal recessive	Achromatopsia (613856)
	*GNB3*	12p13.31	Beta subunit of transducin	Autosomal recessive	Stationary night blindness (617024)^a^
	*PDE6A*	5q33.1	Alpha subunit of rod PDE	Autosomal recessive	RP (613810)^a^
	*PDE6B*	4p16.3	Beta subunit of rod PDE	Autosomal dominant	Stationary night blindness (163500)^a^
				Autosomal recessive	RP (613801)^a^
	*PDE6G*	17q25.3	Gamma subunit of rod PDE	Autosomal recessive	RP (613582)^a^
	*PDE6C*	10q23.33	Subunit of cone PDE	Autosomal recessive	Achromatopsia;^b^ cone dystrophy (613093)
	*PDE6H*	12p12.3	Subunit of cone PDE	Autosomal recessive	Achromatopsia (610024)^b^
	*CNGA1*	4p12	Alpha subunit of rod cyclic nucleotide‐gated channel	Autosomal recessive	RP (613756)^a^
	*CNGB1*	16q21	Beta subunit of rod cyclic nucleotide‐gated channel	Autosomal recessive	RP (613767)^a^ (some patients also have olfactory dysfunction)
	*CNGA3*	2q11.2	Alpha subunit of cone cyclic nucleotide‐gated channel	Autosomal recessive (also digenic with *CNGB3*)	Achromatopsia (216900); cone dystrophy
	*CNGB3*	8q21.3	Beta subunit of cone cyclic nucleotide‐gated channel	Autosomal recessive (also digenic with *CNGA3*)	Achromatopsia (262300); cone dystrophy
Shut‐off	*GRK1*	13q34	Rhodopsin kinase	Autosomal recessive	Oguchi disease (613411);^a^ RP

*SAG*	2q37.1	Arrestin	Autosomal dominant	RP (620228)

Autosomal recessive	Oguchi disease (258100);^a^ RP (613758)

*ARR3*	Xq13.1	Cone arrestin	X‐linked	High myopia (301010) (limited to females)^b^

*RGS9*	17q24.1	Regulator of G‐protein signalling 9	Autosomal recessive	Bradyopsia (608415)

*RGS9BP*	19q13.12	Regulator of G‐protein signalling 9 binding protein	Autosomal recessive	Bradyopsia (620344)
Calcium feedback	*GUCY2D*	17p13.1	Retinal guanylate cyclase 2D	Autosomal dominant	Cone or cone‐rod dystrophy (601777)
				Autosomal recessive	LCA (204000); RP; night blindness (204000)^a^
	*GUCA1A*	6p21.1	Guanylate cyclase activating protein 1A	Autosomal dominant	Cone or cone‐rod dystrophy (600364)
	*SLC24A1*	15q22.31	Solute carrier family 24 (sodium/potassium/calcium exchanger) member 1	Autosomal recessive	Stationary night blindness (613830);^a^ RP^a^

*Note:* The genes *GNB1* and *GNB5* also encode proteins involved in phototransduction, but associated diseases are syndromic with neurological involvement. The genes *GUCA1B* and *GUCA1C*, which encode alternative forms of guanylate cyclase activating protein, have been associated with IRD, but the evidence is not currently as strong as for *GUCA1A*.

Abbreviations: PDE, phosphodiesterase; RP, retinitis pigmentosa.

^a^ Conditions with congenital night blindness.

^b^ Conditions frequently associated with myopia.

In the retina, phototransduction also takes place in intrinsically photoreceptive ganglion cells, which express melanopsin. However, diseases affecting retinal ganglion cells are outside the scope of this review.

## 2 Outline of Rod and Cone Phototransduction

Lamb recently reviewed the cellular and molecular basis of rod and cone phototransduction, including the evolution of phototransduction genes [1]. The grouping of genes in Table 1 (first two columns) into those involved in activation, shut‐off and calcium feedback broadly follows his classification. Below, the processes involved will be outlined briefly, and the reader is referred to several recent reviews for greater detail [1, 2, 3, 4, 5, 6, 7].

### 2.1 Rod Phototransduction Activation

In the dark, an inward current flows into the rod outer segment via CNG cation channels (maintained in their open state by binding cyclic guanosine monophosphate, cGMP). This depolarises the cell, resulting in continual glutamate release at the synaptic terminal (where the photoreceptor synapses with bipolar cells). Light photons, captured by the chromophore that is bound to rhodopsin, initiate the phototransduction cascade, which leads to a reduction in cytoplasmic cGMP. Rhodopsin is present in high concentration in the rod outer segment discs, likely forming dimers or oligomers. The vitamin A‐derived chromophore in both rods and cones is 11‐*cis*‐retinal, which is isomerised by a photon of light into all‐*trans*‐retinal. This molecule is converted back to 11‐*cis*‐retinal via the visual cycle (recently reviewed) [8, 9, 10], which conventionally involves the retinal pigment epithelium and which will not be reviewed here, although variants in genes involved in this pathway are also an important cause of retinal disease [10, 11, 12].

Photoisomerisation of the chromophore causes rhodopsin to change to an active state. Rhodopsin is a G‐protein coupled receptor, and the chromophore effectively switches, upon photoisomerisation, from acting as an antagonist, or inverse agonist, to acting as an agonist. The G‐protein that is activated by rhodopsin is transducin. The latter, once activated, binds to and activates phosphodiesterase‐6 (PDE). The three proteins are present in the disc membrane (~700 to 800 discs are present within a typical mammalian rod outer segment), and so are in close proximity, with diffusion occurring effectively in a two‐dimensional surface, enhanced by relatively high membrane fluidity. Activated PDE hydrolyses cGMP, reducing the cytoplasmic concentration, thus resulting in the closure of CNG channels in the plasma membrane. The consequent reduction in inward cation current hyperpolarises the cell, reducing glutamate release at the synapse.

Relevant genes encoding the above proteins that will be discussed in this review include the following: *RHO* encodes rhodopsin; *GNAT1* encodes the alpha subunit of rod transducin; *PDE6A*, *PDE6B* and *PDE6G* encode subunits of rod PDE; *CNGA1* and *CNGB1* encode alpha and beta subunits of the rod CNG channel (each channel is a heterotetramer, comprising three alpha subunits and one beta subunit).

Fully dark‐adapted rods show extremely high sensitivity, able to respond reproducibly to the absorption of a single photon, through a combination of factors including high concentrations of phototransduction proteins, very high thermal stability of the chromophore, coupled with high efficiency of photoisomerisation, and very low levels of background electrical noise. Having a multi‐step cascade of reactions in the process of phototransduction allows for high sensitivity (each activated rhodopsin can activate multiple G‐proteins; each PDE hydrolyses many cGMP molecules) and scope for multiple sites of adaptation (each activation stage has shut‐off reactions, whose kinetics alter in different light environments, including through calcium‐mediated feedback). As background light intensity increases, adaptation mechanisms reduce rod sensitivity, allowing them to avoid saturation in low backgrounds.

In 1992, Lamb and Pugh modelled the kinetics of phototransduction, explicitly considering the kinetics of each activation stage, deriving a single final expression that showed a remarkably close fit to suction pipette recordings of changes in outer segment current from single photoreceptors in response to light flashes of a range of intensities [13]. This model has been applied widely, including to the a‐wave of the electroretinogram (ERG). Direct application to the ERG a‐wave, however, was later shown to have limitations; Robson and Frishman subsequently developed a more comprehensive model, taking into account current flows in other parts of the photoreceptor [14, 15]. However, the initial stages of that model still reflect outer segment phototransduction, essentially recapitulating the Lamb and Pugh model.

### 2.2 Cone Phototransduction Activation

In cone photoreceptors, the phototransduction cascade proceeds with similar steps. The chromophore is the same, but the opsins confer specific wavelength sensitivity profiles, so that the wavelength of peak absorption differs for the 3 types of cone opsin, which all differ from the peak absorption of rhodopsin. Long and medium wavelength sensitive cones (L‐ and M‐cones) make up the majority of the cone population, conferring high spatial acuity as well as serving red–green discrimination. Short wavelength sensitive cones (S‐cones) are in the minority and are absent from the central fovea. Instead of outer segment discs, cones have membranous sacs (with similar longitudinal spacing to rod discs) that are contiguous with the plasma membrane. Cones have lower maximal sensitivity compared with rods but show quicker recovery from light flashes and adaptation over a much wider range. Thus, cones mediate vision at brighter light levels (most light levels encountered in modern daily living), conferring high acuity (due to high foveal density and a low cone‐to‐bipolar cell ratio) and temporal resolution; rods, in contrast, have higher sensitivity, mediating vision in dimmer light levels, but rod‐mediated vision has poorer spatial and temporal resolution.

Relevant genes encoding proteins in the activation phase of cone phototransduction include the following: *OPN1LW*, *OPN1MW and OPN1SW* encode the L‐, M‐ and S‐cone opsins respectively; *GNAT2* encodes the alpha subunit of cone transducin; *GNB3* encodes the beta subunit of transducin; *PDE6C* and *PDE6H* encode subunits of cone PDE; *CNGA3* and *CNGB3* encode alpha and beta subunits of the cone CNG channel (as in rods, each channel is a heterotetramer, comprising 3 alpha subunits and 1 beta subunit). The Lamb and Pugh (1992) model demonstrated applicability to light‐evoked changes in cone outer segment current. It was also subsequently applied to the cone‐driven ERG a‐wave, but much of the light‐adapted a‐wave has been shown to originate in cone‐driven OFF‐bipolar cells, thus not solely reflecting photoreceptor outer segment current flows [16, 17, 18].

### 2.3 Recovery of the Light Response in Rods and Cones

Each activation step in the phototransduction cascade has shut‐off mechanisms. Some of these are shown in Figure 1 and will be briefly mentioned here. Rhodopsin kinase, a G‐protein receptor kinase, phosphorylates rhodopsin, quenching its activity; further inactivation occurs by the binding of arrestin. Regulator of G‐protein signalling‐9 (RGS9) has a role in shutting down the activity of transducin, so it is no longer able to activate PDE. cGMP levels are replenished by guanylyl cyclase (GC), which is itself activated by guanylyl cyclase activating protein (GCAP). Some of the shut‐off mechanisms are modified by intracellular calcium levels. GCAP, for example, is inhibited by calcium. As calcium levels fall during light exposure (due to reduced entry via CNG channels, but continued extrusion largely via the sodium/calcium, potassium exchanger, NCKX), inhibition of GCAP is reduced, thus enhancing the restoration of cGMP levels by GC [19].

Relevant genes encoding the above proteins are as follows: the G‐protein receptor kinase is encoded by *GRK1* in rods and *GRK7* in cones (which also express some *GRK1*); *SAG* encodes rod arrestin and *ARR3* encodes cone arrestin; *RGS9* encodes the RGS9 protein and *RGS9BP* encodes its binding protein; *GUCY2D* encodes guanylyl cyclase and *GUCA1A* encodes GCAP; other forms of photoreceptor GC and GCAP are encoded by other genes. NCKX is encoded by the *SLC24A1* gene in rods and by *SLC24A2* and *SLC24A4* in cones; of these, *SLC24A1* is associated with an IRD.

### 2.4 Light and Dark Adaptation

The human visual system can adapt to maintain responsiveness over a billionfold range of background light intensities, with much of this adaptation occurring within the photoreceptors themselves. As background light levels increase, the amplitude and duration of photoreceptor light responses diminish, largely via an acceleration in response recovery, allowing the rods to avoid saturation (namely the complete shut‐off of outer segment photocurrent). However, at moderate background intensities, rod outer segment current is fully abolished, rendering the rods electrically unresponsive to light. Cone photoreceptors are responsive at these light levels. They can similarly adapt, but over a much larger range, such that even at extremely high light levels, they are able to maintain up to 50% of the outer segment current at steady state [20]. This is achieved through a combination of accelerated recovery and reduction in the quantal catch due to pigment bleaching. Some longer term adaptation may also occur through translocation of phototransduction proteins between the inner and outer segments, altering their concentration in the outer segment [21]. This may also underlie the more recently described ability of rods to avoid saturation even at very high background light intensities [22, 23].

Whilst light adaptation, described above, is rapid, dark adaptation, the recovery in visual sensitivity following prolonged bright light exposure, is slow. Rods can take over 40 min to recover full sensitivity following a near‐total bleach of photopigment. The reduced sensitivity is not simply a consequence of the reduced quantal catch. In rods, free opsin, unbound to 11‐*cis*‐retinal, appears to be able to activate transducin and therefore the phototransduction cascade, shutting off the outer segment current, thus rendering the rod electrically unresponsive to light. It is not until the free opsin is ‘removed’ by binding to 11‐*cis*‐retinal that the rods regain sensitivity. In cones, however, unbound opsin does not appear to have the same effect; the cone outer segment current can recover within 100 ms following a near‐total steady‐state bleaching exposure, meaning bright flashes can be detected almost instantly, but full sensitivity takes several minutes to recover, following a time course similar to the regeneration of chromophore [20]. Human retinal dark adaptation kinetics, and techniques to track them electrophysiologically, have been discussed in more detail in a recent review [9].

## 3 Monogenic Diseases Primarily Affecting Rod Phototransduction Activation

The majority of diseases considered in this section are forms of rod‐cone dystrophy, synonymous with retinitis pigmentosa (RP), a progressive degeneration of rod, and later cone, photoreceptors. In those in which there is bi‐allelic loss of function (the *recessive* disorders associated with these genes), one important clinical feature is the frequent presence of congenital or early onset night blindness (since rod phototransduction is impaired from the start, preceding any later rod cell death), and this can help distinguish these disorders from other genetic causes of RP, where night blindness is not congenital. Rarely, some of the genes can be associated with stationary night blindness.

### 3.1 Rhodopsin

*RHO* is one of the genes most frequently associated with autosomal dominant retinitis pigmentosa and is the first gene in which RP‐associated variants were found [24, 25, 26, 27, 28]. More than 150 disease‐associated variants have been reported since. Pathogenic variants can cause disease by several different mechanisms, with multiple classification systems in the literature and a spectrum of disease severity. Athanasiou et al. grouped variants into seven categories, using biochemically studied characteristics, based on key mechanisms, such as altered trafficking to the outer segment, misfolding, altered or constitutive transducin activation, or efficiency of dimerization [29]. Many variants remain unclassified, and there is likely also to exist some overlap between mechanisms. The commonest reported disease‐associated variant in North America is p.(Pro23His). This variant appears less common in other cohorts; in the United Kingdom, one relatively frequently encountered variant is p.(Pro347Leu), associated with more severe disease.

Those variants which result in constitutive activation of transducin give rise to congenital night blindness (since the rod outer segment current will be shut off, reducing or abolishing their electrical responsiveness to light). Other genotypes might give rise to later onset night blindness (as the rods degenerate). The degeneration can be sectoral in distribution, mainly involving the inferior retina, and in some cases can be non‐progressive. Variants in *RHO* have been associated with autosomal dominant congenital stationary night blindness (CSNB), although it is possible that in some cases the disease might not be fully stationary, but very slowly progressive.

Autosomal recessive *RHO*‐associated retinitis pigmentosa is rarer than dominant *RHO*‐associated RP. Here, night blindness will be congenital or early onset, and the disease is more severe than in autosomal dominant *RHO*‐associated RP. Usually, in the context of recessive disease, the risk of a patient's children being affected is very low (in the absence of consanguinity). However, in patients with bi‐allelic variants in *RHO*, the specific variants need to be considered. Some variants might also act to cause disease dominantly, and so advice to patients should be informed by a consideration of what has been previously reported regarding each variant as well as the family history. For example, the p.Glu150Lys variant has been reported homozygously in patients with autosomal recessive *RHO*‐associated RP [30]. However, a knock‐in mouse model suggests that this variant might also lead to a milder degeneration heterozygously [31].

Although pathogenic *RHO* variants give rise to RP (including sectoral RP), one particular variant has been reported to give rise to patchy atrophy that resembles primary retinal pigment epithelium (RPE) degenerations (e.g., similar to that seen in choroideremia) rather than RP: this variant was reported as p.(Met207Lys) [32] but personal communication with the authors confirmed the variant to be p.(Met216Lys).

### 3.2 Rod Transducin

#### 3.2.1 GNAT1

Transducin, a heterotrimeric peripheral membrane protein (Gαtβγ), is cardinal for the first amplification step in the phototransduction cascade (Figure 1). In rods, the α‐subunit of transducin is encoded by *GNAT1*. A limited number of missense variants in *GNAT1* that affect the GTP/GDP‐binding domains and/or the nuclear localisation signal have been implicated in autosomal dominant CSNB (p.Gly38Asp, p.Ile52Asn, p.Gln200Glu) and autosomal recessive CSNB (p.Asp129Gly) [33, 34, 35]. The p.Gly38Asp variant was the cause of dominant CSNB in the large ‘Nougaret’ pedigree [34]. More recently, nonsense variants leading to a truncated protein (p.Gln302* and p.Cys321*) have been linked to autosomal recessive rod‐cone dystrophies [36, 37].

#### 3.2.2 Other Rod Transducin Subunits

*GNB1* encodes the beta subunit of transducin in rods. This gene has been associated with an autosomal dominant neurodevelopmental disorder (MIM 616973). There have been two reports of RP occurring in cases of the *GNB1*‐associated neurodevelopmental syndrome [38, 39]. *GNGT1* encodes the gamma subunit of rod transducin. To date, this gene has not been associated with an IRD.

### 3.3 Rod Phosphodiesterase (PDE)

PDE6 is highly concentrated in the internal membranes of photoreceptors, where it regulates cytoplasmic cGMP levels in the outer segments of rods and cones [40]. Perturbations of cGMP levels disrupt phototransduction and elevated cGMP levels can trigger photoreceptor apoptosis [41]. In the dark, PDE exhibits low catalytic activity as the *α* and *β* catalytic subunits of PDE are bound to *γ* subunits; PDE is activated when transducin binding removes the inhibition of the *γ* subunits [42].

#### 3.3.1 *PDE6A* and *PDE6B*

Both the *α* and *β* subunits encoded by *PDE6A* and *PDE6B* are required for full enzymatic activity [43]. Variants in the genes that encode these subunits are a relatively common cause of autosomal recessive RP (arRP) [44]. Natural history studies indicate the symptom onset is typically in childhood, and in the majority of patients, the disease has a slowly progressive course [45, 46]. Of note, patients harbouring biallelic null variants in the *PDE6A* gene tend to have the youngest age of diagnosis as well as the most rapid loss of ellipsoid zone [45].

A heterozygous variant in *PDE6B*, p.His258Asp, has been reported as the cause of autosomal dominant CSNB in a large Danish pedigree (the ‘Rambusch pedigree’) [47]. More recently, another heterozygous variant, p.Tyr314Cysfs*50, has been reported to cause the same disorder, similarly by giving rise to constitutive activation [48].

#### 3.3.2 PDE6G

Pathogenic variants in *PDE6G* are rare, and only a handful of cases have been reported to date. All affected individuals were found to have severe early‐onset RP [49, 50]. A Pde6g‐deficient mouse model recapitulates the phenotype in affected patients, showing very early photoreceptor degeneration [44].

### 3.4 Rod CNG Channels

#### 3.4.1 CNGA1

Mutations in the *CNGA1* gene, which encodes the alpha subunit of the rod cGMP‐gated channel, were first identified in families with arRP in 1995 [43]. The prevalence of mutations that account for arRP varies by geographic region [43, 51, 52]. A significant number of *CNGA1* variants are copy number variants or frameshift variants and are predicted to have a deleterious effect on key functional domains or impair membrane trafficking [53, 54].

#### 3.4.2 CNGB1

Mutations in the *CNGB1* gene account for approximately 0.4%–1% of arRP cases [55]. The *CNGB1* gene locus encodes several splice variants. The full‐length protein is exclusively expressed in the retina, but a shorter gene transcript that lacks the glutamic acid‐rich protein (GARP) domain is expressed in the sensory neurons of the olfactory epithelium and is involved in olfactory signal transduction. Eliciting symptoms of olfactory dysfunction (though not present in all patients) is helpful, as it not only suggests *CNGB1* as a potential causative gene but also might suggest that the mutations affect the channel domain, not the GARP domain [56, 57]. Most of the missense variants in the *CNGB1* gene are located in the channel domain, with two ‘hot spots’ at the level of the transmembrane domains and at the level of the cyclic nucleotide‐binding domain [55]. In contrast, most of the variants that affect the GARP domain of the protein are nonsense, frameshift or affect splicing.

## 4 Monogenic Diseases Primarily Affecting Cone Phototransduction Activation

### 4.1 Cone Opsins

Blue cone monochromacy (BCM) is a rare X‐linked disorder characterised by a marked reduction or absence of L‐opsin and M‐opsin expression (encoded by the neighbouring *OPN1LW* and *OPN1MW* genes), impairing L‐ and M‐cone function [58, 59]. Several genetic mechanisms have been described: (i) a deletion involving the red‐green locus control element that prevents expression of genes in the array is associated with the most severe phenotype, (ii) a 2‐step process in which the number of cone opsin genes in the array has been reduced to one due to a non‐homologous recombination, followed by an inactivating mutation, commonly p.(Cys203Arg), in the remaining gene and (iii) specific combinations of single nucleotide polymorphisms on exon 3 that result in aberrant splicing [60].

Compared with patients with achromatopsia (detailed in the next section), the S‐cones are normal, and patients might have less severely affected visual acuity, usually ranging between 20/80 and 20/200 [59]. Electrophysiology testing can distinguish these two cone dysfunction syndromes. In achromatopsia, the single‐flash LA 3 ERG is usually undetectable; in BCM, a small amplitude b‐wave appearing at approximately 50 ms is suggestive of an S‐cone system origin and can be confirmed with an extended S‐cone ERG protocol [61, 62].

BCM patients are also myopic, and the condition has phenotypic overlap with Bornholm Eye Disease, where specific variants in exon 3 of the opsin genes give rise to cone dysfunction, dichromacy and high myopia [63].

Variants affecting only the L‐opsin or M‐opsin gene can give rise to X‐linked red‐green colour deficiencies, namely deuteranopia and protanopia, with less severe variants giving rise to deuteranomaly and protanomaly. Variants affecting the S‐opsin gene, *OPN1SW*, give rise to tritanopia or tritanomalous vision, impairing blue‐yellow discrimination. Individuals with tritanopia have normal visual acuity (given the foveal centre does not contain S‐cones even in healthy individuals).

### 4.2 Cone Transducin

#### 4.2.1 GNAT2

Bi‐allelic variants in *GNAT2* (which encodes the alpha subunit of cone transducin) cause achromatopsia [64, 65]. This was the third gene to be associated with achromatopsia, after *CNGA3* and *CNGB3* (described below). Other genes associated with achromatopsia include *PDE6C* and *PDE6H* (also described below) and *ATF6*. Patients have congenital loss of cone function (resulting in impaired visual acuity, loss of colour vision, photoaversion, usually associated with nystagmus), with intact rod function; achromatopsia is also known as rod monochromacy. Patients with *GNAT2*‐associated achromatopsia can have relative preservation of cone structure on retinal imaging [66].

#### 4.2.2 GNB3

The β subunit of the G protein, encoded by *GNB3*, not only modulates cone transducin function but also cone and rod ON bipolar cell signalling. Consequently, biallelic pathogenic variants lead to a dual anomaly in visual processing with defective ON bipolar signalling (giving rise to congenital night blindness) and variable cone sensitivity deficits demonstrable on electrophysiological testing [67, 68].

#### 4.2.3 Other Cone Transducin Subunits

*GNGT2* encodes the gamma subunit of cone transducin. To date, this gene has not been associated with an IRD.

### 4.3 Cone PDE

*PDE6C* and *PDE6H* respectively encode the catalytic and γ‐subunit of cone cGMP PDE [69, 70]. While *PDE6C* accounts for a minority of cases of achromatopsia in Europe (< 2%), variants in this gene are a leading cause of achromatopsia in Japan and Korea [71, 72]. Deep phenotyping has shown that while affected individuals have typical symptoms of achromatopsia, they have a distinct natural history and clinical features. They usually have high myopia; foveal hypoplasia is often absent; there can be progressive vision loss accompanied by the development of macular atrophy [69, 73, 74, 75].

A particular nonsense variant in *PDE6H* has been reported homozygously as a rare cause of incomplete achromatopsia, although a case of complete achromatopsia has also been reported in association with the same variant [76].

### 4.4 Cone CNG Channels

In achromatopsia, impairment of cone‐mediated visual function gives rise to a characteristic clinical constellation of reduced vision (typically 20/200), loss of colour discrimination, photophobia, hemeralopia and pendular nystagmus [77]. Although the fundus often appears normal, optical coherence tomography may variably reveal the presence of foveal hypoplasia and/or an optical gap, sometimes with foveal atrophy, while electrophysiological testing demonstrates loss of photopic function with preservation of scotopic function [78, 79, 80]. The complete form is characterised by a lack of function in all three cone types; an incomplete form occurs when there is residual function of one or more cone types, permitting relatively better visual acuity and colour vision [81].

The most common genetic cause of achromatopsia differs by ethnicity [59, 71, 72]. *CNGB3* is the major locus for achromatopsia in patients of European origin or descent, and truncation variants predominate; the p.(Thr383Ilefs*) is a frequent founder mutation [74, 82]. In contrast, *CNGA3* variants are a prevalent cause of achromatopsia in Chinese and Korean patients, with the majority arising from missense changes [74]. It is recognised that digenic triallelic inheritance can occur: this involves biallelic *CNGB3* variants (commonly c.1208G>A) and an additional monoallelic *CNGA3* pathogenic variant [83, 84].

Adaptive optics has permitted cellular resolution in vivo retinal imaging for patients with *CNGA3‐* and *CNGB3‐*related achromatopsia; longitudinal studies have demonstrated that the foveal cone mosaic shows little change over time [85, 86]. The relatively intact fovea in some patients and potentially large treatment window make achromatopsia an attractive treatment target.

## 5 Monogenic Diseases Affecting Recovery From the Light Response and Other Processes

### 5.1 Shut‐Off of Activated Rhodopsin

#### 5.1.1 *GRK1* and *SAG*

Oguchi disease is a rare form of autosomal recessive CSNB arising from biallelic pathogenic variants in *GRK1* or *SAG*. Impaired shut‐off of activated rhodopsin results in the rods remaining hyperpolarised (due to prolonged phototransduction activation) and electrically unresponsive to light (hence impairing rod‐mediated vision); the rods are effectively light‐adapted. The rods can regain their sensitivity after prolonged (several hours) dark adaptation [87]. A striking clinical feature is the golden hue of the fundus that disappears with prolonged dark adaptation and reappears with light exposure (Mizuo‐Nakamura phenomenon) [88]. The correlate of the sheen on optical coherence tomography appears to be a merging of the outer retinal hyperreflective bands (ellipsoid zone line and retinal pigment epithelium) [89]. Even in the light‐adapted state, the sheen has been noted to disappear in the presence of subretinal fluid or after exposure to bright short‐wavelength illumination, suggesting that, in some cases at least, the sheen is dependent on sufficient quantities of 11‐cis‐retinal‐bound rhodopsin [90, 91].

Electroretinography shows preserved LA responses but subnormal dark‐adapted responses (undetectable DA 0.01 and negative DA10, with subnormal a‐wave, largely reflecting the dark‐adapted cone system response); the a‐wave amplitude on DA10 recordings increases following several hours of dark adaptation [9].

The two genes encode proteins which quench phototransduction sequentially: G‐protein‐coupled receptor kinase 1 (GRK1) first phosphorylates light‐activated rhodopsin (R*), which then forms a complex with arrestin (also known as S‐antigen, SAG) [87, 92].

Variants in *GRK1* are postulated to affect phosphorylation of light‐activated rhodopsin through three mechanisms: (i) a loss of protein due to nonsense‐mediated decay; this stems from a frameshift or nonsense variant in all exons except the last, (ii) disruption of the kinase activity because of a change in protein shape and dynamics; this is typically caused by a missense variant and (iii) mislocalisation of the protein due to disruption of the extreme C‐terminus responsible for anchoring GRK1 to the rod outer segment; this arises from a frameshift in the final exon [93].

Of note, variants in *SAG* are also linked to autosomal dominant or recessive RP [87, 94]. There are different founder mutations: in the Japanese population, c.1147delA is linked to both Oguchi disease and RP; in Hispanic families, c.440G>T is a dominant variant linked to toxic protein misfolding [95, 96]. Variants in *GRK1* can also be associated with progressive retinal degeneration.

### 5.2 Shut‐Off of Activated Cone Opsins

*GRK7* encodes the cone G‐protein receptor kinase, but has not so far been linked to disease. Cone arrestin is encoded by *ARR3*.

#### 5.2.1 ARR3

The *ARR3* gene located on the X‐chromosome encodes cone arrestin (also known as X‐arrestin or arrestin‐4). Cone arrestin shares 49% homology with rod arrestin; it inactivates phosphorylated opsins and prevents cone signals. The M/L cones have cone arrestin only, while S cones have both cone and rod arrestin [97, 98]. Variants in this gene are linked to X‐linked dominant early‐onset high myopia [97, 99]. Intriguingly, females (who will carry the variant heterozygously) are affected rather than males (who carry the variant hemizygously). A possible explanation is hypothesised in Section 9.

### 5.3 G‐Protein Shut‐Off

#### 5.3.1 *RGS9* and *RGS9BP*

Bradyopsia (from the Greek words for slow vision) is linked to biallelic mutations in genes critical to enabling rapid recovery of phototransduction [100]. There is limited ability of transducin to hydrolyse GTP due to a genetic defect in *RGS9* (regulator of G‐protein signalling 9) or *RGS9BP* (RGS9‐binding protein, also known as G‐protein signal 9‐anchoring protein) [101, 102]. Patients with this stationary cone dysfunction syndrome often have delayed dark and light adaptation, photophobia, difficulty tracking moving objects, and visual acuity that is better in reduced luminance conditions [102, 103]. Unlike some of the other cone dysfunction syndromes (rod or S‐cone monochromacy or oligocone trichromacy), affected individuals usually do not exhibit nystagmus and have normal colour vision [102].

This condition is one of a few with pathognomonic electroretinogram abnormalities and had initially been described as ‘Prolonged Electro‐Retinal Response Suppression’ [103, 104]. The DA 10 ERG shows a normal response to the first flash but the subsequent responses are attenuated with a standard interstimulus interval of 20 s. This normalises when the interstimulus interval is considerably increased [103]. Additionally, an extended ISCEV protocol incorporating a scotopic red flash ERG reveals good dark‐adapted cone function. This, combined with the encouraging observation on high‐resolution AO imaging that the cone photoreceptor mosaic is intact, suggests that the cones are potentially amenable to rescue [100].

*GNB5* encodes a protein that forms complexes with RGS (regulator of G‐protein signalling) proteins. It is expressed in the brain and in the retina (in photoreceptors, bipolar cells and other cell types). Bi‐allelic variants are associated with an autosomal recessive neurodevelopmental syndrome (MIM 617173 and 617 182). In one patient, ERG findings showed retinal signalling defects with some features resembling bradyopsia and other features attributable to impairment of bipolar cell signalling [105].

### 5.4 Recovery of Cyclic GMP Levels

#### 5.4.1 GUCY2D

The *GUCY2D* gene accounts for up to 20% of all patients with autosomal recessive Leber Congenital Amaurosis (arLCA) and up to 25% of all autosomal dominant cone‐rod dystrophy (adCRD) [106, 107]. It is also rarely associated with selective rod‐predominant dysfunction (autosomal recessive) [108]. The encoded enzyme, retinal guanylate cyclase 1 (RETGC‐1), restores cytoplasmic cGMP needed for CNG channels to reopen and photoreceptors to return to a dark‐adapted state [109].

*GUCY2D* was the first gene linked to arLCA (LCA1) and this usually arises from null mutations (frameshift, nonsense or splicing) [109]. LCA1 is one of the most severe forms of LCA; vision can be no light perception at the initial examination [110]. Despite severe visual deficits, the structural integrity of photoreceptors appears relatively well preserved, suggesting that restoration of functional RETGC‐1 to photoreceptors has the potential to improve vision [110].

The dominant variants linked to CRD are clustered in exon 13, of which codon 838 is a notable mutation hotspot (pathogenic variants include p.(Arg838Cys), p.(Arg838Ser), p.(Arg838His), p.(Arg838Gly) and p.(Arg838Ala)) [109, 111]. The mutated protein remains active, keeping cGMP levels high and CNG channels open; this toxic gain of function heightens the influx of Na^+^ and Ca^2+^ in photoreceptors, which induces apoptosis [112]. Unlike LCA1 patients who are often hypermetropic, *GUCY2D*‐related adCRD patients are often myopic [106, 108].

#### 5.4.2 GUCA1A

The *GUCA1A* gene is highly expressed in cones and rods, where it encodes the guanylyl cyclase activating protein (GCAP1). This protein is essential for maintaining photoreceptor functional integrity by regulating RETGC activation and cGMP regeneration [113]. GCAP1 activates RETGC but is inhibited by calcium. Pathogenic variants in *GUCA1A* are associated with autosomal dominant cone or cone‐rod dystrophy as well as autosomal dominant macular dystrophy [114]. Very rarely, an RP phenotype has been reported [115].

Approximately 20 disease‐associated variants have been reported. The p.(Tyr99Cys) variant was the first to be identified, with several others reported since [113]. These variants appear to cluster in regions where they affect calcium sensitivity (abolishing calcium‐mediated inhibition). The p.(Pro50Leu) variant was mistakenly associated with disease in an early report, but was later shown to be likely to be a benign polymorphism, with disease in the reported family being attributable to a pathogenic variant in *RPGR* [116, 117].

The genes *GUCA1B* and *GUCA1C* also encode GCAPs, expressed in rods and cones, or mainly cones, respectively. There have been some reports suggesting an association between variants in these genes and IRDs, but so far the evidence is not as strong as for *GUCA1A*: the reported missense variant in *GUCA1B* appears to occur more commonly than would be expected for a fully penetrant autosomal dominant rare disease variant (it is present in 0.7% of East Asian alleles in the genomes aggregation database version 4.1, https://gnomad.broadinstitute.org/ accessed 27 Jan 2025); a variant in *GUCA1C* has been reported as a possible association in a single report [118, 119].

### 5.5 Other Proteins

*SLC24A1* encodes the sodium/calcium potassium exchanger that is expressed in the rod outer segment [120]. This permits calcium extrusion, and it is hypothesised that complete or even partial loss of this ion exchange function could lead to abnormal levels of intracellular Ca2+ concentrations, disrupting rod function [120]. Biallelic variants in this gene were associated with autosomal recessive CSNB in 2010 [120], with additional families reported subsequently [121]. However, the gene has also been associated with autosomal recessive RP, suggesting that in addition to congenital night blindness, mild, progressive degeneration can occur [122, 123, 124].

The cone sodium/calcium potassium exchanger is encoded by *SLC24A2*, which has not, to date, been associated with disease. Several other proteins involved in rod and cone phototransduction (some mentioned in prior sections) have also not yet been associated with disease.

## 6 Phenotypic Features Across Disease Groups

This review has discussed the associated IRDs in summary form. For detailed natural history data, including longitudinal changes in retinal imaging, the reader is referred to several prior publications (and their associated citations) [45, 46, 55, 59, 66, 73, 125, 126]. In the following section, some common phenotypic features will be highlighted, followed by some selected examples of findings from imaging and electrophysiology.

As expected, diseases primarily affecting rod phototransduction give rise to night blindness as the initial symptom, whilst those primarily affecting cone phototransduction usually give rise to acuity loss and photoaversion, also affecting colour discrimination. (Although *GNB3* is more expressed in cones than in rods, the night blindness can arise from its expression in ON‐bipolar cells.) The autosomal recessive diseases affecting rod phototransduction usually give rise to congenital night blindness with subsequent retinal degeneration (distinct from many other forms of RP where night blindness is often not congenital). Some rare autosomal dominant forms give rise to stationary night blindness.

Whilst many of the diseases discussed are progressive, some are largely stationary (including Oguchi disease and achromatopsia), with some genes associated with both progressive and stationary disease. It is possible that some diseases deemed stationary are, in reality, very slowly progressive. *GUCY2D* is associated with perhaps the largest range of phenotypes: bi‐allelic variants can give LCA, but also a disease selectively affecting rod function; dominant variants give rise to a cone or cone–rod dystrophy.

Myopia is a prominent feature of some of the disorders, including blue cone monochromacy, Bornholm eye disease, *PDE6*‐associated achromatopsia (but not achromatopsia associated with *CNGA3* or *CNGB3*) and *ARR3*‐associated disease (in females). Most of the diseases described do not have manifestations outside the eye (attributable to the highly photoreceptor‐specific expression of such genes), but *CNGB1*‐associated retinitis pigmentosa can also be associated with olfactory dysfunction (arising from the gene's expression in olfactory receptors).

### 6.1 Features on Retinal Imaging

**FIGURE 2:**
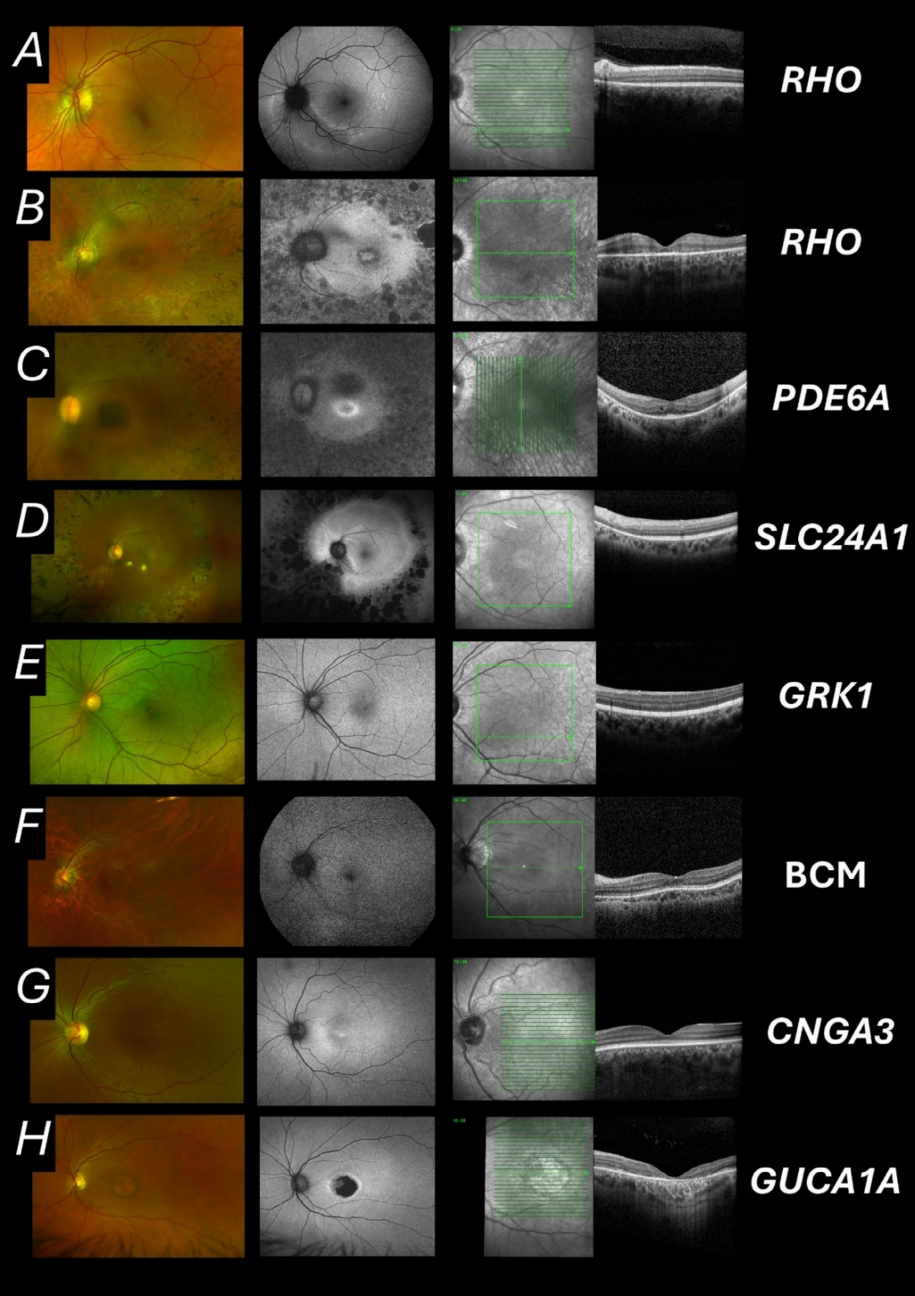
Examples of retinal imaging in patients with monogenic disease associated with some phototransduction genes, primarily affecting rod (A–E) or cone (F–H) phototransduction. In all rows, the leftmost panel shows pseudocolour imaging (Optos plc, Dunfermline, UK); the next panel shows autofluorescence imaging (532 nm obtained with Optos in all rows except A and F, where this was 488 nm obtained with Spectralis, Heidelberg Engineering, Heidelberg, Germany); the right panels show spectral domain optical coherence tomography (OCT) images (Spectralis) with the corresponding infrared reflectance image showing the position of the OCT scan (green arrow). All images are of the left eye, but right eye findings were similar. Some conditions can be associated with a spectrum of phenotypes and progression; examples shown are not exhaustive. (A) 38 year old female with the *RHO* variant p.Glu341Lys heterozygously. A sectoral distribution of abnormal autofluorescence is visible corresponding with loss of outer retina seen in the nasal part of the OCT scan (taken in the inferior macula). (B) 34‐year old male with the *RHO* variant p.Pro347Leu variant heterozygously. This patient has more severe disease, consistent with the severity of this variant. (C) 37‐year old male with the *PDE6A* variant c.1408‐2A>G homozygously. The autofluorescence image shows the hyperautofluorescent ring seen in many forms of RP. (D) 59 year old male with the *SLCA24A1* variant p.Met252Valfs*2 homozygously. The hyperautofluorescent ring is very wide, with most of the macula preserved (small area of outer retinal loss visible on nasal aspect of OCT scan taken far from the foveal centre), despite congenital or early onset night blindness. (E) 26‐year old female with *GRK1‐*associated Oguchi disease (variants: p.(Arg393Ter) and p.(Leu449Arg)). The left panel shows the characteristic sheen. The OCT image (acquired aged 28) shows relative proximity of the outermost hyperreflective bands, thought to correlate with the sheen. (F) Male patient with BCM (pseudocolour image acquired aged 20; autofluorescence and OCT images acquired aged 24). (G) 21‐year old female with *CNGA3‐*associated achromatopsia. (H) 59‐year old male with autosomal dominant *GUCA1A*‐associated cone dystrophy (harbouring the p.Tyr99Cys variant heterozygously).

Figure 2 shows examples of retinal imaging findings in several of the diseases discussed. The upper panels (A–E) show examples in diseases affecting rod phototransduction. *RHO*‐associated RP can vary markedly in severity depending largely on the particular variant (with a relatively mild and a more severe example shown). Panel C shows the characteristic hyperautofluorescent ring that is usually seen in RP irrespective of genetic cause, highlighting the border between preserved retina (within the ring) and degenerated retina [127]. *SLC24A1* has been associated with normal fundus findings (autosomal recessive stationary night blindness), but variants can give rise to RP. This is very mild (very large hyperautofluorescent ring as seen in Figure 2D), despite the night blindness being congenital or early onset. Oguchi disease (Figure 2E) is characterised by a striking fundus sheen (usually correlating with relative merging of hyperreflective outer retinal bands on OCT), distinguishing it from other causes of stationary night blindness (including *RDH5*‐associated fundus albipunctatus, which can have a similar ERG phenotype, but which demonstrates white dots on clinical examination).

The lower panels (F–H) show examples in diseases affecting cones. BCM can present similarly to achromatopsia, but the former will only affect males (being X‐linked) and may show preservation of S‐cone ERGs, with affected individuals having some element of colour discrimination (as S‐cones and rods differ in wavelength sensitivity). Patients with BCM are also usually myopic. Clinically, the fundus may appear unremarkable in achromatopsia and BCM, although foveal atrophy may be present; OCT usually shows outer retinal abnormality and ERGs facilitate the diagnosis and help distinguish between these and some other forms of cone system dysfunction. For example, *CABP4*‐associated disease can present with similar symptoms to achromatopsia (photophobia, impaired vision and a stable course), but the ERG phenotype is quite different (with an electronegative dark‐adapted ERG seen in *CABP4*‐disease). In *GUCA1A*‐associated disease (Figure 2H), the condition is autosomal dominant, affecting central vision, but visual impairment is not congenital, developing later in life with variable degrees of macular outer retinal degeneration and generalised cone system dysfunction.

### 6.2 Electroretinography

Most, if not all, of the conditions listed affect the full‐field ISCEV (International Society for Clinical Electrophysiology of Vision) standard ERG [128]. Those affecting rod phototransduction will primarily affect dark‐adapted responses, and those affecting cone phototransduction will primarily affect the light‐adapted responses. Importantly, as phototransduction is affected, the a‐wave will be subnormal in most of these conditions (since the a‐wave arises largely from the hyperpolarisation of photoreceptors that results from phototransduction shutting the outer segment CNG channels). This is helpful in distinguishing it from other genetic diagnoses. ERGs can be particularly helpful in evaluating the extent and locus of retinal dysfunction, particularly when fundus imaging is normal or shows mild abnormalities. Rarely, ERG changes can be pathognomonic. Extended ERG protocols can be helpful in some conditions, such as the S‐cone ERG protocol (helpful in BCM and enhanced S‐cone syndrome) and the dark‐adapted red flash (helpful in *RGS9* and *RGS9BP*‐associated disease) [61, 129]. The extended protocol for the derivation and analysis of the strong flash rod‐isolated ERG a‐wave (subtracting out the cone‐driven portion of the bright‐flash response) can be helpful to quantitatively assess diseases affecting rod phototransduction [130].

Some of the conditions discussed entail a phenotype of CSNB. Oguchi disease is recognisable from the fundus sheen. However, the fundus can appear unremarkable in rare forms of autosomal dominant CSNB associated with variants in *RHO*, *GNAT1* or *PDE6B*, that lead to constitutive activation of rod phototransduction. These will give rise to a subnormal dark‐adapted bright flash (DA10) a‐wave (‘Riggs‐type’ CSNB), which might have a negative waveform (discussed below). This is in contrast to other types of CSNB where the DA10 a‐wave is of normal amplitude, but the b‐wave is selectively and profoundly reduced (electronegative ERG, ‘Schubert‐Bornschein‐type’ CSNB) [9]. The latter type of CSNB involves a site of impairment that is post‐phototransduction and can be subdivided into ‘complete’ CSNB (associated with variants in *NYX* when X‐linked, or *TRPM1*, *GRM6*, *GPR179* or *LRIT3* when autosomal recessive) or ‘incomplete’ CSNB (associated with variants in *CACNA1F* when X‐linked, or *CABP4* when autosomal recessive). Incomplete CSNB usually entails photophobia in addition to, or instead of, nyctalopia (particularly in disease associated with *CABP4*). Where night blindness is acquired rather than congenital, with normal fundus imaging, other, non‐genetic, aetiologies including vitamin A deficiency and melanoma‐associated retinopathy should be considered.

In RP, rod‐driven ERGs are more affected than cone‐driven ERGs, but severe rod system dysfunction (due to degeneration) will usually entail some cone system dysfunction. However, in autosomal recessive RP associated with rod phototransduction proteins, it is possible, particularly in mild disease, to observe severe rod system dysfunction (here owing to rods not functioning rather than having degenerated) with relatively little cone system involvement. Thus, the ERGs can appear similar to those seen in rare autosomal dominant CSNB (Riggs‐type as discussed above), autosomal recessive diseases such as Oguchi disease or fundus albipunctatus, and in Vitamin A deficiency (which selectively affects rod function unless advanced or severe). Such ERGs with normal cone system function have been reported in a case of *CNGB1*‐associated RP [131].

**FIGURE 3:**
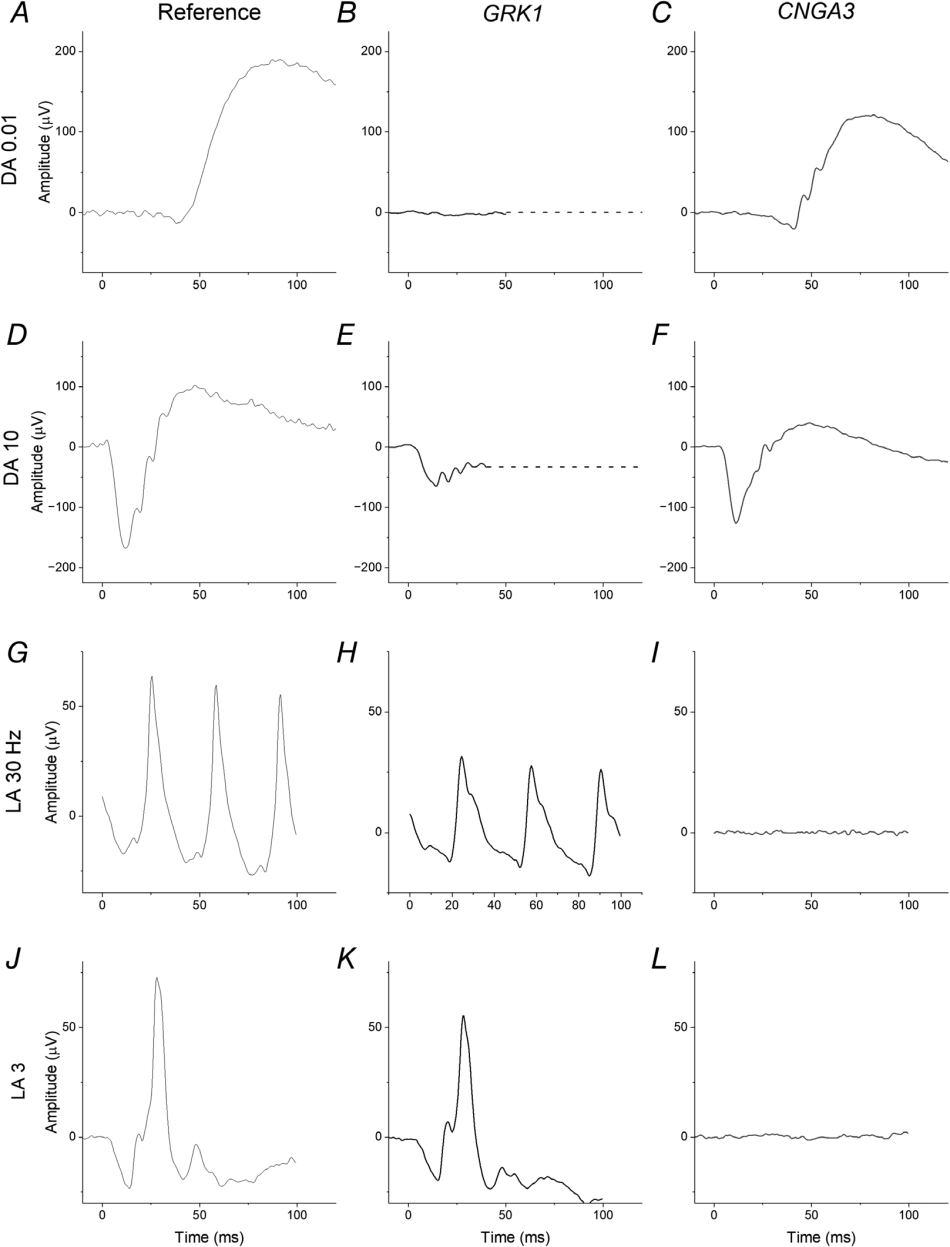
Examples of standard ERG responses in selective congenital impairment of rod or cone phototransduction. The left‐hand column shows healthy control responses. These were averaged from a cohort of healthy adults but are also representative of the waveforms obtainable from a healthy individual. The middle column shows ERGs from an individual with GRK1‐associated Oguchi disease. The right‐hand column shows ERGs from an individual with *CNGA3*‐associated achromatopsia. (A–C) DA 0.01 ERG; (D–F) DA 10 ERG; (G–I) LA 30 Hz ERG; (J–L) LA 3 ERG. DA, dark‐adapted; LA, light‐adapted. The numbers 0.01, 10, and 3 refer to flash strength in photopic cd s m^−2^.

Figure 3 shows examples of ISCEV standard full‐field ERGs obtained in two selected conditions: one entailing complete loss of rod photoreceptor responses, with intact cone signalling, and the other with complete loss of cone responses, with intact rod signalling. The left‐hand column shows healthy responses averaged from a healthy cohort for comparison [132].

The middle column shows responses from a patient with *GRK1*‐associated Oguchi disease. Here, the DA 0.01 response is undetectable, indicating rod system dysfunction. The DA10 ERG shows a markedly reduced a‐wave, indicating impairment in rod phototransduction (activated rhodopsin has not been switched off by rhodopsin kinase and hence the rods are already hyperpolarised). The b‐wave is further reduced, but this should not be interpreted as representing additional inner retinal pathology. Here, the ERG is largely from the dark‐adapted cone system, whose bright‐flash response often has a smaller b‐wave in relation to the a‐wave, but this shape cannot usually be seen in individuals with healthy rod function [133]. The light‐adapted responses are well preserved. Such ERGs can be observed in the other causes of selective loss of rod function described above. (In Oguchi disease, and in some other conditions including fundus albipunctatus, the DA10 a‐wave may normalise following prolonged dark adaptation.)

The right column of Figure 3 shows responses from an individual with *CNGA3*‐associated achromatopsia. As cone CNG channels are dysfunctional, the light‐adapted responses are undetectable. The dark‐adapted ERGs are largely preserved. The mild possible reduction in the DA10 a‐wave (panel F) can be attributed to the loss of the cone‐driven component of the DA10 a‐wave (the very component preserved in the patient with Oguchi disease, shown in panel E).

## 7 Prevalence of Disease Associated With Phototransduction Genes in a UK‐Based Cohort

**FIGURE 4:**
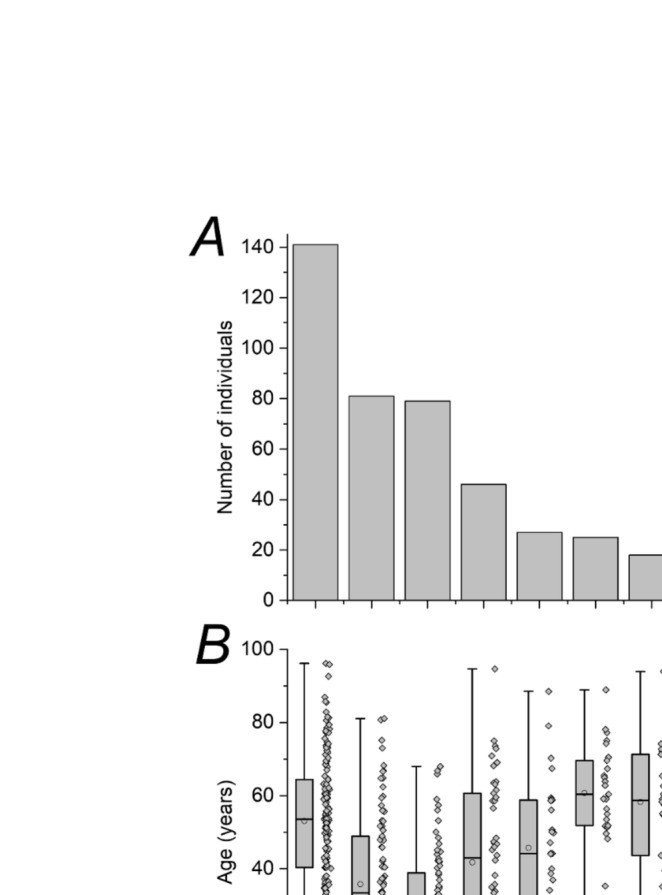
Numbers and ages of individuals with disease associated with phototransduction genes in a large UK‐based cohort. (A) Numbers of individuals with disease associated with each gene (for genes with more than five individuals). (B) Boxplots and data showing the distribution of ages of individuals (ages are at time of data extraction, not age of onset). Medians are shown by horizontal lines; means are shown by circles; whiskers are maximum and minimum values. Individual ages for all patients are also shown for each gene.

The Genetics Service at Moorfields Eye Hospital in London oversees a very large cohort of IRD patients, representing the largest single‐centre cohort of IRD patients with a confirmed molecular genetic diagnosis. A recent publication reported the spectrum of variants in 4415 patients with positive molecular diagnoses [26]. Of these patients, 456 (10.3%) had disease‐associated variants in genes discussed in this review. The upper panel of Figure 4 shows numbers of patients by gene (for genes with more than five patients). The top five genes were *RHO*, *CNGB3*, *CNGA3*, *GUCY2D* and *PDE6B* (with 141, 81, 79, 46 and 27 affected patients respectively).

The lower panel plots the range of patient ages for each gene (these were ages at the time of the study rather than ages of disease onset). The lowest median ages were for *CNGA3* and *CNGB3*, reflecting the congenital onset of the associated condition (achromatopsia). A large range was seen for *RHO*, reflecting the large numbers as well as the range of severity and ages of onset. A large range was also seen for *GUCY2D*, with the youngest patients likely reflecting the LCA phenotype. For *GUCA1A*, however, no children were observed, reflecting the later onset of the cone dystrophy. Of the recessive RP‐associated genes, *CNGB1* showed the highest mean age, likely reflecting the relatively mild phenotype in comparison to other RP genotypes, despite the congenital night blindness. *SLC24A1* can be associated with even milder RP, but is not shown as fewer than five patients had *SLC24A1*‐associated retinopathy.

It can also be helpful to identify the most common disease‐associated variants in particular cohorts: these can be compared between cohorts from different geographical locations, revealing founder effects, and can also yield insights into genotype–phenotype correlation. Table 2 lists, for reference, the most frequent variant for each of the 5 most frequently encountered phototransduction genes in this cohort. For dominantly inherited disease, where a particular gain of function mechanism might be required to bring about disease, some pathogenic variants tend to occur more frequently, and this is seen particularly in autosomal dominant *GUCY2D*‐associated disease, where variants affecting codon 838 appear to predominate. For *RHO*‐associated dominant disease, where several different mechanisms might lead to disease, many different variants are seen. In this cohort, the commonest disease‐associated *RHO* variant was p.(Pro347Leu), contrasting, for example, with US‐based cohorts where the p.(Pro23His) variant is more frequently seen. In recessive diseases, often arising from loss of protein function, there are usually large numbers of possible disease‐associated variants observed. Interestingly, however, for *CNGB3*‐associated disease, one particular allele accounted for more than 70% of disease‐associated alleles.

**TABLE 2:** Most prevalent pathogenic variants for each of the five most frequently encountered phototransduction IRD‐associated genes in a UK‐based cohort.

Gene	Mode of inheritance in this cohort	Number of patients	Number of disease‐associated alleles in cohort	Most prevalent disease‐associated allele		Frequency of most prevalent allele (% of all disease‐associated alleles for that gene)
				Transcript	Variant	
*RHO*	AD	141	141	NM_000539.3	c.1040C>T, p.(Pro347Leu)	20 (14%)
*CNGB3*	AR	81	162	NM_019098.5	c.1148del, p.(Thr383IlefsTer13)	116 (72%)
*CNGA3*	AR	79	158	NM_001298.3	c.1641C>A, p.(Phe547Leu)	23 (15%)
*GUCY2D*	AD/AR	46	67	NM_000180.4	c.2512C>T, p.(Arg838Cys)	11 (16%)
	AD only	27	27	NM_000180.4	c.2512C>T, p.(Arg838Cys)	11 (41%)
*PDE6B*	AR	27	54	NM_000283.4	c.1107+3A>G	5 (9%)

*Note:* Numbers of alleles reflect the total number of disease‐associated alleles (rather than numbers of unique variants). Modes of inheritance: AD, autosomal dominant; AR, autosomal recessive. For *RHO*, AR inheritance is also seen very rarely; for *PDE6B*, AD inheritance is also seen very rarely. These modes of inheritance were not observed in this cohort. *GUCY2D* can be associated with both AD and AR IRDs (both of which were observed in this cohort). The commonest *GUCY2D* pathogenic variant observed was p.(Arg838Cys), which is associated with AD cone or cone‐rod dystrophy. Another variant affecting the same codon (also associated with AD cone or cone‐rod dystrophy), p.(Arg838His), was observed almost as frequently: It occurred 10 times (accounting for 15% of all disease‐associated *GUCY2D* alleles, and for 37% of all *GUCY2D* alleles associated with AD disease).

## 8 Gene‐Specific Therapies on the Horizon

Several comprehensive reviews of IRDs that include a summary of gene and RNA‐based clinical trials have recently been published [134, 135]. In Table 3, we provide a precis of some gene replacement trials listed on the clinicaltrials.gov website that target phototransduction gene‐related IRDs and are either actively recruiting or have reported outcomes. These are for autosomal recessive disease. So far, many have been reported only in abstract form, and it remains challenging to predict which, if any, are likely to translate into approved treatments. Results from 3 of the trials have been formally published in peer‐reviewed journals: these have related to therapies for *CNGA3*‐associated achromatopsia [136, 142] (with 3‐year results published), *CNGB3*‐associated achromatopsia (6 month results published) [137] and *GUCY2D*‐associated LCA. The *GUCY2D* Phase I/II study safety and efficacy 12‐month data were published in the *Lancet* in September 2024 [141], although some preliminary findings were also reported in earlier publications [138, 139, 140]. The results appear to support further investigation as part of a Phase 3 trial.

**TABLE 3:** Summary of gene therapy trials listed on clinicaltrials.gov which relate to phototransduction gene‐related IRDs.

Gene	Disease	Clinicaltrials.gov identifier	Phase	Therapy under investigation	Reported results in publications or meeting abstracts
*PDE6A*	Retinitis pigmentosa	NCT04611503	I/II	Subretinal AAV8 gene therapy (rAAV.hPDE6A)	Nine patients treated. Postoperative decline in VA occurred in seven patients; this was mild and resolved by month 3 in five patients but two patients had moderate to severe VA loss that did not resolve by month 12 (Reichel et al., ARVO Abstract 782, 2023)
*PDE6B*	Retinitis pigmentosa	NCT03328130	I/II	Subretinal AAV2/5 gene therapy (HORA‐PDE6B)	17 patients treated. Untreated eyes declined as per disease's natural course; treated eyes showed stabilisation in most tests including VA, VF, microperimetry, mobility test, and horizontal line of the ellipsoid zone (Ducloyer et al., ARVO Abstract 2134, 2023)
*CNGA1*	Retinitis pigmentosa	NCT06291935	Ib	Intravitreal AAV2 gene therapy (AAV2.NN‐CNGA1)	First patient dosed in Apr 2023; estimated completion is Dec 2025
*CNGA3*	Achromatopsia	NCT02610582	I	Subretinal AAV8 gene therapy (rAAV.hCNGA3)	Nine patients treated. Functional benefits that were noted in the treated eye at year 1 were persistent throughout the following visits at years 2 and 3 [136]
	Achromatopsia	NCT02935517	I/II	Subretinal AAV gene therapy (rAAV2tYF‐PR1.7‐hCNGA3)	24 patients treated. Reported to be safe and well‐tolerated in children up to and including second highest dose of 1.1e12 vg/mL, and in adults up to and including the highest dose of 3.2e12 vg/mL (Iannaccone et al., ARVO abstract 2829, 2022)
	Achromatopsia	NCT03758404	I/II	Subretinal AAV gene therapy (AAV.CNGA3)	11 patients enrolled (results posted on clinicaltrials.gov, last updated Dec 2022)
*CNGB3*	Achromatopsia	NCT03001310	I/II	Subretinal AAV8 gene therapy (AAV8‐hCARp.hCNGB3)	23 patients treated (11 adults and 12 children). Favourable changes for colour vision (*n* = 6/23), photoaversion (*n* = 11/20), and vision‐related quality‐of‐life questionnaires (*n* = 21/23) [137]
	Achromatopsia	NCT02599922	I/II	Subretinal AAV gene therapy (rAAV2tYF‐PR1.7‐hCNGB3)	31 patients treated. Reported to be safe and well‐tolerated in children up to and including second highest dose of 1.1e12 vg/mL, and in adults up to and including the highest dose of 3.2e12 vg/mL (Iannaccone et al., ARVO abstract 2829, 2022)
*GUCY2D*	Autosomal recessive LCA	NCT03920007	I/II	Subretinal AAV5 gene therapy (ATSN‐101)	15 patients treated. Well‐tolerated and improvement in visual function; the greatest effect was observed with high‐dose ATSN‐101 (1·0 × 10^11^ vg/eye) [138, 139, 140, 141]

Abbreviations: AAV: adeno‐associated virus; MLMT: multi‐luminance mobility testing; VA: visual acuity; VF: visual field.

For autosomal dominant disease, methods other than replacement of the defective gene are likely to be required. One trial that includes patients with *RHO*‐associated disease (NCT05203939) entails delivery, not of the *RHO* gene, but of nuclear hormone receptor‐based gene modification therapy, inspired by mouse models in which Nr2e3 gene therapy ameliorated degeneration in diverse genetic causes of RP [143]. The human trial includes patients with *RHO* and *NR2E3*‐associated disease, but preliminary results have only been published so far in abstract form (Lam et al., ARVO Abstract 406, 2024). Another trial (PRODYGY, NCT05748873) entails delivery of a gene expressing rod‐derived cone viability factor in patients with advanced RP associated with *RHO*, *PDE6A* or *PDE6B*. Preliminary safety results from the first 3 patients have been published in abstract form (Martel et al., ARVO Abstract 3091, 2024). Other gene‐specific methods in autosomal dominant disease could include gene silencing and gene editing approaches, but these are mostly in relatively early stages of investigation. Patients with disease associated with phototransduction genes are also likely to be candidates for future restorative or regenerative therapies that are not gene specific.

Other modalities of therapy might be applicable in future for specific genetic causes. For example, dominant cone dystrophy associated with gain‐of‐function in *GUCY2D* or *GUCA1A* entails abnormally high cGMP levels. Attempting to drive down cGMP levels by light exposure could theoretically slow degeneration and represents a potential avenue for exploration. In transgenic mice carrying the p.Tyr99Cys variant in GCAP‐1 (encoded in humans by *GUCA1A*), light exposure ameliorated degeneration [144]. A prior study also demonstrated rescue in such mice by constitutive activation of rod phototransduction using a rhodopsin transgene containing the p.Gly90Asp variant [145]. Thus, understanding the dynamics of phototransduction could yield avenues for exploration of future therapies.

## 9 Conclusions

Monogenic diseases associated with rod and/or cone phototransduction represent a significant proportion (~10% in our cohort) of IRDs. Understanding the molecular pathway of phototransduction and its kinetics can yield a better understanding of these conditions as well as guide avenues of exploration for future therapies. Recognising several distinctive phenotypic features can improve diagnostic speed and accuracy, which will become increasingly important as gene‐specific therapies emerge. Genetic testing is now frequently conducted via large gene panels or whole genome sequencing, often yielding variants of uncertain significance in many genes. Correlation with the phenotype (which includes the clinical and family history, multimodal imaging and electrophysiology), informed by an understanding of phototransduction mechanisms, will facilitate correctly identifying which of these variants are likely to be causative of disease or identifying the likely genes in which causative variants might have been missed.

It is also likely that some currently unsolved IRD cases will arise from variants in genes that have yet to be identified as being associated with disease. Some of the genes that encode phototransduction proteins but have not yet been associated with IRDs might be potential candidates (including, for example, *GNGT1*, *GNGT2*, *GRK7*, *SLC24A2* and others).

Where proteins form part of the same pathway or the same channel, it is likely that variants in other genes will modify the phenotype: this is shown by the digenic/tri‐allelic cases of *CNGB3*/*CNGA3*‐associated disease [83] but the principle is likely to apply more broadly in modifying disease severity. Advances in genetic pathway analyses and also in techniques of structurally and functionally evaluating the retina, including mathematical modelling of ERG waveforms, are likely to yield further insights [15].

More common variants in these genes might modify other aspects of retinal structure and physiology or pathophysiology. A recent genome‐wide association study (GWAS) of photoreceptor sub‐structure in a population‐based cohort highlighted several genetic loci; interestingly, these included variants in or near some phototransduction‐associated genes (*GNAT2*, *RHO*, *GRK1*, *SAG*), and this could have relevance to non‐monogenic diseases of outer retinal structure, including age‐related macular degeneration [146].

IRDs are often associated with refractive error [147, 148, 149]. Myopia is increasing in prevalence worldwide; its causes are multifactorial and rarely monogenic [150]. However, understanding which IRDs predispose to myopia or hyperopia, and to what degree, could yield insights into molecular mechanisms driving myopia. A recent study of axial lengths in molecularly proven IRDs included some of the conditions discussed here: axial lengths were significantly longer in BCM and Bornholm Eye Disease; *CNGA3*‐ and *CNGB3*‐associated achromatopsia did not show an increased axial length [148]. Why some forms of cone dysfunction predispose to myopia (including *PDE6C*‐associated disease), whilst others do not, is of interest. Variants in *ARR3* (the X‐linked gene encoding cone arrestin) lead to high myopia in females rather than males. It is possible that the existence of populations of cones terminating their light responses with different kinetics (as would occur in heterozygous females) is more myopia‐genic than a situation in which all of the cones have slower shut‐off (which presumably occurs in males hemizygous for the same *ARR3* variant) [125].

Many phototransduction genes have also been highlighted in a large recent meta‐analysis of refractive error GWAS data [151], supporting the hypothesis that more common variants in these genes might subtly alter phototransduction and contribute to myopia. Thus, greater understanding of phototransduction could yield important insights not just into monogenic retinopathies, but also into more common eye diseases.

## Conflicts of Interest

O.A.M. has participated in an Advisory Board meeting for Janssen.

## Data Availability

Data sharing is not applicable to this article as no new data were created or analyzed in this study.
